# Saxitoxin in Alaskan commercial crab species

**DOI:** 10.1371/journal.pone.0330132

**Published:** 2025-09-03

**Authors:** Patricia A. Tester, Bruce Wright, Richard Wayne Litaker, Patryce McKinney, William C. Holland, Steven R. Kibler, Mark W. Vandersea

**Affiliations:** 1 Ocean Tester, LLC, Beaufort, North Carolina, United States of America; 2 Knik Tribe, Palmer, Alaska, United States of America; 3 CSS Inc. Under contract to National Oceanic and Atmospheric Administration, National Centers for Coastal Ocean Science, Beaufort Laboratory, Beaufort, North Carolina, United States of America; 4 Department of Environmental Conservation, Division of Environmental Health, Environmental Health Laboratory, Anchorage, Alaska, United States of America; 5 National Oceanic and Atmospheric Administration, National Centers for Coastal Ocean Science, Beaufort Laboratory, Beaufort, North Carolina, United States of America; Lusofona University of Humanities and Technologies: Universidade Lusofona de Humanidades e Tecnologias, PORTUGAL

## Abstract

Paralytic shellfish poisoning (PSP) is a pervasive human health concern associated with subsistence, recreationally and commercially harvested Alaskan shellfish. PSP is caused by saxitoxins (STX), a family of structurally similar neurotoxins produced by the marine microalgae *Alexandrium catenella* (formerly *A. fundyense*). These toxins accumulate in filter-feeding shellfish such as clams, mussels and oysters. While PSP is commonly associated with consuming bivalves, toxic STX levels can also be found in crab viscera (crab butter). The first cases of PSP from consuming Dungeness crab viscera (*Metacarcinus magister*) were reported in 1992. Although this incident and others did not involve commercially harvested crab, they did impact management of the Dungeness crab fishery in Alaska. Current regulations in southeast Alaska permit the sale of whole Dungeness crab, whereas those in the Kodiak Archipelago must have their viscera removed post-harvest to prevent PSP. This study examines the impacts of STXs and current regulations on the Alaskan crab fishery, with a focus on Dungeness crab. Data on commercial landings and the value of harvested Dungeness crab and processed products showed that regulations to protect human health, combined with market forces over the past 30 years, have shifted the fishery’s focus toward Dungeness crab products without viscera. The study also presents time series data on STX concentrations in Dungeness crab from 1992 to 2023, along with maps indicating collection locations and their associated toxicity levels. The same data for King crab (*Paralithodes* or *Lithodes* spp.) and Tanner (Snow) crab (*Chionoecetes* spp.) are included to assess the prevalence of STX in these commercially harvested species. Further, a preliminary analysis suggests regional variations in the toxicity of *A. catenella* strains could affect regional shellfish toxicity.

## 1. Introduction

Paralytic shellfish poisoning (PSP) is a persistent health threat in Alaska. This illness is caused by potent neurotoxins, known as saxitoxins (STX), which are produced by the marine microalga *Alexandrium catenella*. These toxins can accumulate in filter feeding molluscan shellfish or crustacean shellfish that prey on other organisms containing STX. Following the discovery of high STX levels in fish collected during a large 1992 mortality event, the Alaska Department of Environmental Conservation (ADEC) conducted extensive toxin testing to quantify STX concentrations in commercially harvested species. This effort included Alaskan shellfish [1]. Results showed that STX in Dungeness crab harvested from the Kodiak and Alaskan Peninsula management regions regularly exceeded the advisory limit of 80 µg STX eq. 100 g viscera^-1^. In contrast, the crab harvested from other management areas either did not exceed the advisory limit or did so infrequently. Based on these findings the Alaska Department of Fish and Game (ADF&G) implemented regulations requiring processors to eviscerate Dungeness crab harvested in the Kodiak management region before sale (Soares in [1]). Dungeness crab from management areas with lower toxicity, such as southeast (SE) AK, were allowed to be sold live. The only exception occurred in 1994 when a large *A. catenella* bloom occurred in SE AK, triggering an 18-day closure of the summer Dungeness crab fishery and a ban was placed on the sale of all live and frozen whole crab products [1].

Although evisceration regulations currently only apply to Dungeness crab from the Kodiak region, Deeds et al. [2] noted that, while not well documented in the scientific literature, the Alaska Department of Environmental Conservation – Environmental Health Laboratory (ADEC-EHL) has observed elevated levels of STXs in viscera from several commercially harvested crab species for years with concentrations exceeding 500 μg STX eq. 100 g viscera^-1^ almost annually in some areas. The potential significance of these observations for human health was highlighted during inquiries made through the ADF&G Subsistence Division Director about the frequency of crab viscera consumption. Emma Pate, the Training Coordinator and Environmental Planner in the Office of Environmental Health at Norton Sound Health Corporation, responded: “Yes, we eat everything but the shells…. That goes for Red King crab, Blue King crab, Tanner crab, there is also a Japanese crab Hanasaki”.

**Fig 1:**
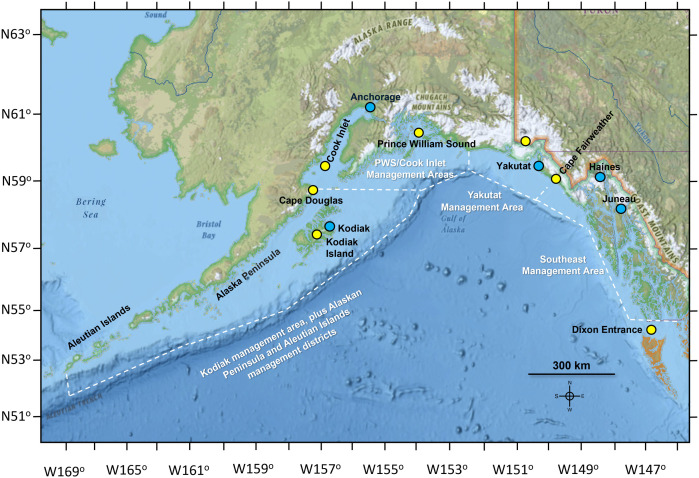
Map of Alaska Department of Fish and Game Management Areas for Dungeness Crab. The Southeast and Kodiak management areas were the focus of this study due to the paucity of data in the other management areas. The Southeast Management Area extends north from Dixon Entrance, the boundary between Canada and the U.S. to Cape Fairweather AK. The Kodiak Management Area located in the western Gulf of Alaska south of Cape Douglas was combined with data from the Alaskan Peninsula and Aleutian Islands for this study (https://www.adfg.alaska.gov/index.cfm?adfg=fishingCommercialByFishery.statmaps). These various management areas were plotted on Esri’s National Geographic Base Map – Sources: National Geographic, Esri, DeLorme, HERE, UNEP-WCMC, USGS, NASA, ESA, METI, NRCAN, GEBCO, NOAA, iPC.

In 2010 a Dungeness crab related PSP case was reported by a Haines, AK fisherman. During this event, after meals of butter clams, three other PSP cases were recorded on Kodiak Island and a fatality occurred in the Juneau area from eating cockles (*Clinocardium* sp.) (Fig 1) [3,4]. These incidents were the result of a coastwide *Alexandrium* bloom. During the spring and summer of that year, increased shellfish toxicity was first observed along the U.S. North Pacific coast starting in Washington [5]. Subsequently, STX toxicity was detected in British Columbia, southeast (SE) AK, the Gulf of Alaska, Kodiak and the Aleutians [6]. Saxitoxin levels peaked in Kodiak by July 2010 in mussels (2,695 µg STX eq 100 g viscera^-1^).

To determine the potential impact of STX concentrations in Dungeness crab and the regulations requiring evisceration of commercially harvested Kodiak crab, on both human health and prices, we compared multiple data sets from the SE AK and Kodiak management aeeas. These inculded how often STX concentrations in Dungeness crab exceeded the regulatory limit for safe consumption, ex-vessel landings (kg) and prices ($) of processed commercial products. between the SE AK and Kodiak management areas. The available STX data spanned the period from 1992 to 2023 and the landings data from 1984 to 2023. To provide context for these findings, the temporal and spatial variations in STX toxicity of Dungeness, Tanner and King crab were also included. Preliminary data on the toxicity of *A. catenella* strains indicated regional variations in toxicity per cell. The higher STX concentrations found in strains from Kodiak may account for the elevated toxicity observed in Dungeness harvested from this management area.

## 2. Background and methods

### 2.1. Data sources

ADEC-EHL Data Set Request and Utilization Agreement Forms were signed to access the STX toxicity data used in this study. Data include saxitoxin concentrations in µg STX eq. 100 g viscera^-1^ of Dungeness, King crab (Red *Paralithodes camtschaticus* as well as some Blue *P. platypus* and Golden *Lithodes aequispinus*), and Tanner (Snow) crab (*Chionoecetes opilio, C. baridi, C. tanneri*). To protect the anonymity of fishing locations in this historic dataset, maps were constructed using the center point of the management area polygon where the catch was recorded (see 2.3). Commercial landings and economic data were obtained from ADF&G staff, website (https://www.adfg.alaska.gov/index.cfm?adfg=fishlicense.coar_buying) and reports identified in the following sections.

Maps throughout this study were created using ArcGIS® software by Esri. The National Geographic Base Map was used for all maps to facilitate comparisons [7]. ArcGIS® and ArcMap™ are the intellectual property of Esri and are used herein under license. Copyright © Esri. All rights reserved. Any errors or deficiencies in reproduction, subsequent analysis, or interpretation; and any statements, findings, conclusions, and recommendations herein do not necessarily reflect the views of ADF&G.

### 2.2. Saxitoxin testing protocols in crustaceans

The protocols for measuring STX concentrations in various Alaskan shellfish species differ. ADEC-EHL performed mandatory testing of commercially harvested molluscan shellfish sold in Alaska using standardized protocols established by the National Shellfish Sanitation Program (NSSP) [8]. When testing crab, only the viscera is tested as studies have shown this is the only tissue where STX accumulates [9,10]. This is of public concern because in some Alaskan communities crab viscera are frequently consumed.

Saxitoxin concentrations in crab viscera were determined using mouse bioassays (MBA) following the AOAC Official Method of Analysis 959.08 or High-Performance Liquid Chromatography (HPLC) known as the Lawrence method [11]. The MBA results were then converted to HPLC STX equivalents 100 g viscera^-1^ as described in [12] to standardize data reporting. The minimum toxicity that can be quantitatively estimated using this approach is approximately 30−32 µg STX eq. 100 g viscera^-1^. Concentrations below this threshold were considered as non-detectable (ND).

### 2.3. Saxitoxin in Alaskan Dungeness crab viscera

To compare STX toxicity differences among crab species, data were grouped into categories based on the percentage of samples with viscera STX levels of < 80, 80−250, 250−500, 500−800 and 800−2,000 µg STX eq. 100 g viscera^-1^. These categories were plotted as STX concentration versus the percentage of total samples. The intervals began with the regulatory limit of 80 µg STX eq. 100 g viscera^-1^ [2]. Time series graphs were used to display all STX values of Dungeness crab viscera from 1992 to 2023 illustrating how sample collection activity varied among different regions over time.

Regional maps indicate where areas of higher toxicity occurred. Samples were assigned the latitude and longitude corresponding to the center point of the statistical area polygon where they were harvested and mapped using Esri’s National Geographic base map in the ArcGIS Online version 10.8.2 [13] (S1 Fig). Since the center point of the management area polygons or statistical chart area polygons were used to provide a point location for the toxicity data, *no assumptions regarding the exact fishing locations should be made*. *Data patterns should be interpreted as regional rather than point specific*. Furthermore, it is important to understand that samples analyzed by ADEC-EHL for STX in commercially harvested crab fisheries were generally taken during pre-season surveys, as sub-samples during the season or because of a reported illness. There was no systematic, area wide, ongoing sampling for STX for any Alaskan crustacean fishery.

### 2.4. Dungeness crab landings and values

Ex-vessel fisheries landings and economic data from the Kodiak and SE AK fisheries management areas for the years 1984–2023 were downloaded from ADF&G websites and annual ADF&G Fishery Management Reports for Dungeness crab [14–16]. Economic data were adjusted for inflation to 2023 U.S. dollar values [17]. These data were used to analyze the potential impact of the evisceration requirements implemented in Kodiak in 1992 on landings and ex-vessel prices of Dungeness crab.

Initially, the annual landings (converted to kg) of Dungeness crab from Kodiak and SE AK from 1984–2023 were plotted simultaneously to establish how specific patterns in ex-vessel landings varied between regions. Data were not available for the Kodiak management area from 2000–2008 because ADF&G policy precluded sharing data on landings or prices when records from fewer than three processors or vessels were available [18]. Annual ex-vessel prices per kg of Dungeness crab harvested in Kodiak and SE AK show how ex-vessel prices received for Dungeness crab varied by area between 1984 and 2023.

### 2.5. Changes in Dungeness crab products from 1984 through 2023

The landings and wholesale values of commercially harvested Dungeness crab were obtained from ADF&G Commercial Operator’s Annual Reports (COAR) from 1984−2023 [19]. The specific products reported include fresh whole, fresh sections, frozen whole, frozen sections, cooked whole and cooked sections. To examine the potential impact of the 1992 evisceration regulation in Kodiak, the data sets were segregated into marketed products containing viscera (live, fresh, frozen and cooked whole) and those which did not contain viscera (fresh, frozen, and cooked sections) [1,20–22]. Time series graphs of total weight of products, annual price kg^-1^ and total value were summed for comparisons of viscera and non-viscera containing products. It should be noted that data on whole and cooked sections were available only since 1998. Despite the data limitations associated with confidentiality considerations, there were sufficient data to identify trends in the amount and price of processed products before and after 1992 when evisceration regulation went into effect for the Kodiak Dungeness crab fishery.

### 2.6. Saxitoxin in Alaskan Tanner and King crab viscera

Unlike Dungeness crab which are captured nearshore, King and Tanner (Snow) crab are often harvested from deeper waters, including the Bering Sea. Since King and Tanner crab are part of a winter fishery and are less frequently sampled for STX in their offshore habitats, the STX data from all management areas for each species were plotted as a single time series consisting of sampling date versus the corresponding STX concentration in the viscera. Toxicity time series plots and toxicity distribution maps of the entire King and Tanner crab STX databases were constructed following the same procedures used for Dungeness crab.

### 2.7. Regional differences in *Alexandrium catenella* cell toxicities

Unpublished data on the STX content of single-cell *Alexandrium catenella* isolates from Kodiak and SE AK collected in association with the work reported by Vandersea et al. [23] were included in this study. These data provided a possible explanation for observed regional differences in the frequency of shellfish exceeding regulatory limits. Briefly, single-cell *A. catenella* isolates were established as described in [23]. Once isolates were established, separate 200 ml cultures were started for toxicity determination. Cell concentrations in each culture were determined from 5 ml aliquots every other day using a Coulter Counter™ (Beckman Coulter Instruments, Indianapolis, Indiana, USA). When each culture reached late exponential phase (~10,000 cell ml^-1^), a 5 ml aliquot was removed and the cells were gently pelleted by spinning at 3,500 rpm in a Thermo Fisher Multifuge X Pro centrifuge (Thermo Fisher, Waltham, Massachusetts, USA) for 10 min and the supernatant was poured off. Pellets were immediately frozen and stored at −80°C. The cell pellets were subsequently extracted by adding 2 ml of methanol/deionized water (80/20) and sonicated for 5 minutes at 60 Hz on ice using a Q Sonica Q500 sonicator (Q Sonica Inc., Newtown, Connecticut, USA). The sonicated cells were then vortexed for 30 s and assayed for STX concentration following the published Eurofins Abraxis ELISA® protocol (Gold Standard Diagnostics, Davis, California, USA). Vortexed aliquots were immediately diluted 1:10 and 1:50 in kit buffer. A five µl aliquot from each dilution was then assayed for STX. The resulting toxicity determinations were expressed as pg STX eq. cell^-1^ and plotted for comparison of the cell toxicity results from isolates collected from the Kodiak and SE AK. A two-tailed T-test for two independent means was performed to determine if the isolates obtained from the SE AK versus Kodiak management areas had significantly different toxicities [24].

## 3. Results

### 3.1. Saxitoxin concentrations in Alaskan Dungeness crab

**Fig 2:**
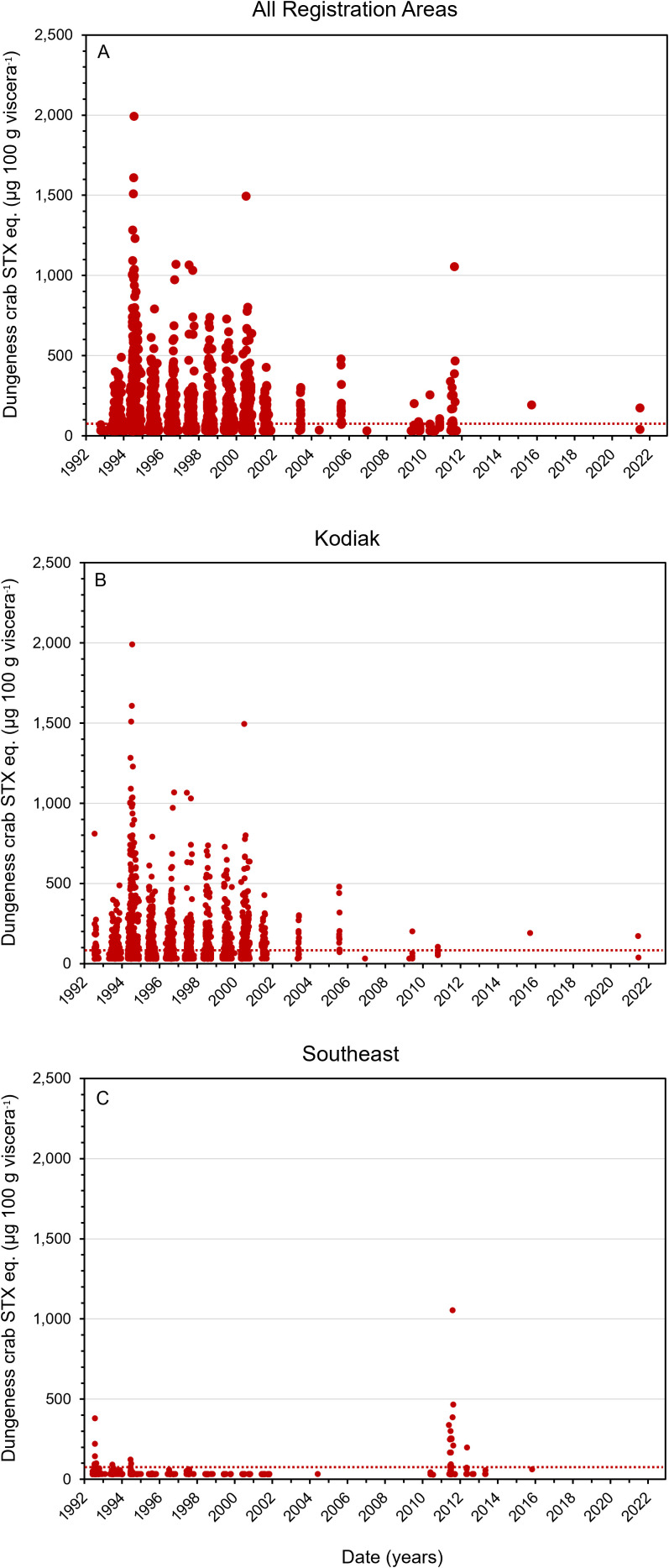
Saxitoxin concentrations in commercially harvested Alaskan Dungeness crab viscera. (A) Saxitoxin data from all management areas from 1992 to 2023 (N = 3,108). (B) Saxitoxin data from the Kodiak management area showing the frequency of samples collected from 1992−2023. N = 2,080. (C) Saxitoxin data from the Southeast Alaska management area showing when samples were collected during the period from 1992−2023. N = 786. The red line indicates the 80 µg STX equivalents 100 g viscera^-1^ regulatory limit for safe harvest of Dungeness crab. The X-axis tick marks indicate 1 January of each year. Data source: Alaska Department of Environmental Conservation – Environmental Health Laboratory.

The time series of STX toxicity in Alaska Dungeness crab commercially harvested between 1992 and 2023 represents all management areas (Fig 2A). Sampling was most intense from 1993 to 2002 when ADF&G was investigating variations in the toxicity of Dungeness crab viscera in different management areas.

**Fig 3:**
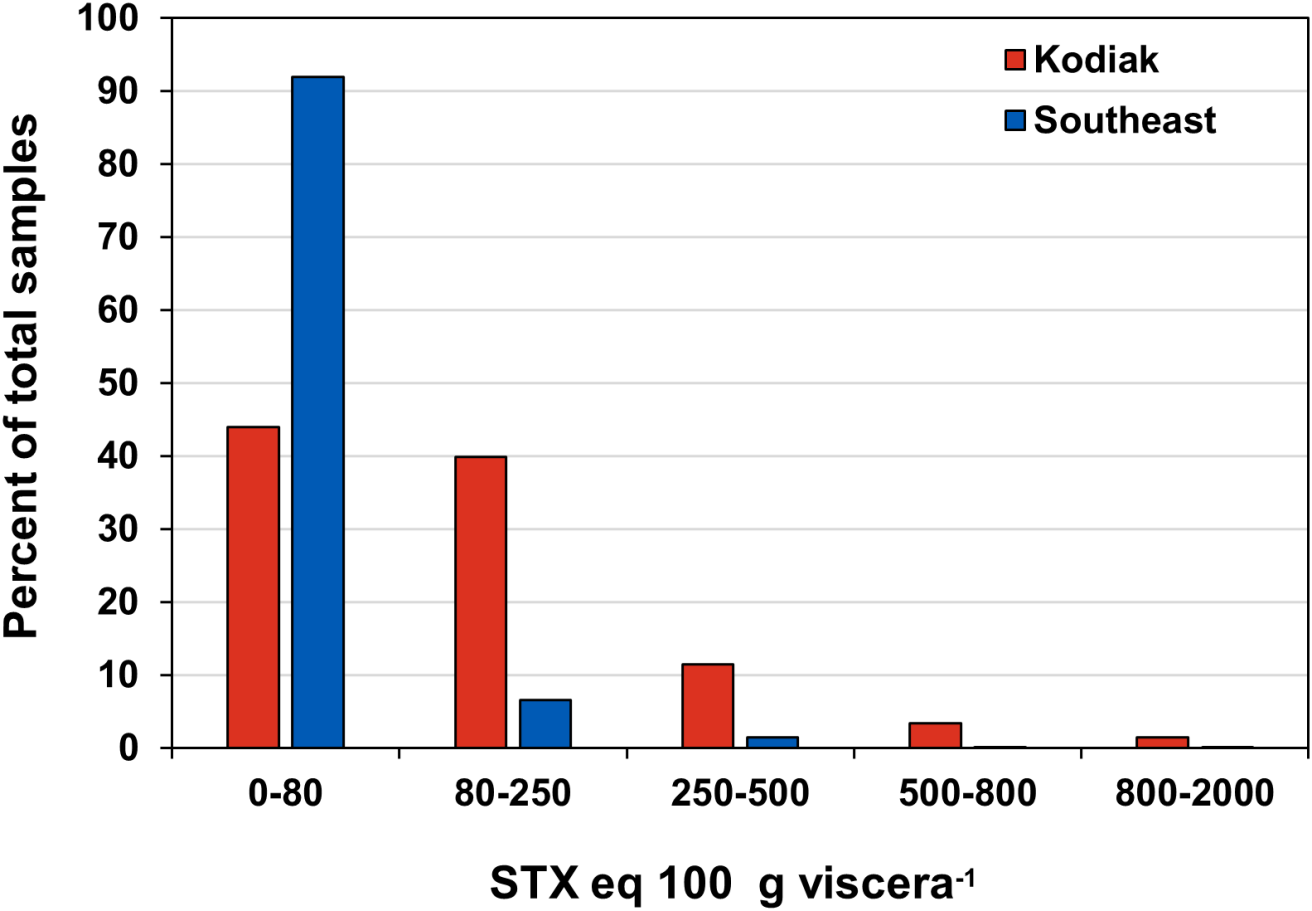
Percentages of saxitoxin contaminated commercially harvested Dungeness crab. The data from Fig 2B and C were converted to percentages of contaminated samples to allow comparison between the Kodiak and Southeast Alaska management areas. Data source: Alaska Department of Environmental Conservation – Environmental Health Laboratory.

**Fig 4:**
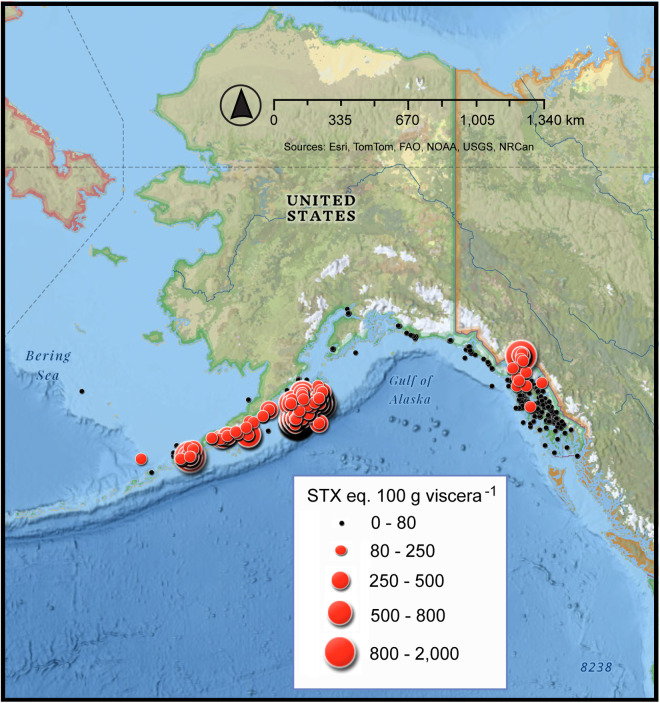
Geographic distribution of saxitoxin concentrations in the viscera of commercially harvested Alaskan Dungeness crab from 1992 to 2023. The size of each dot on the map is proportional to the STX concentration. The STX data were obtained from the Alaska Department of Environmental Conservation – Environmental Health Laboratory. These data were plotted Esri’s National Geographic Base Map – Sources: National Geographic, Esri, DeLorme, HERE, UNEP-WCMC, USGS, NASA, ESA, METI, NRCAN, GEBCO, NOAA, iPC.

The viscera of Dungeness crab from the Kodiak region often exceeded the established 80 µg STX eq. 100 g viscera^-1^ advisory limit (Figs 2B,3). In SE AK the toxicity exceeded the regulatory level less frequently, but concentrations between 200 and 1,000 µg STX eq. 100 g viscera^-1^ were observed (Figs 2C,3). Overall, 56.1% of the viscera samples of Dungeness crab collected from the Kodiak area exceeded the regulatory limit compared with 8.1% in SE AK (Fig 3). Not only was toxicity in the Kodiak fishery more frequent but the highest levels of toxicity were also found there (Fig 4). Sampling in different shellfish management areas was most intense during 1992–1993. During that time the contrast between the Cook Inlet, Prince William Sound and Yakutat management areas, versus the SE and Kodiak was pronounced. None of the Cook Inlet, Prince William Sound and Yakutat crab contained STX concentrations above regulatory limit (Figs 1,4) (Table 1).

**Table 1:** Percentage of saxitoxin positive samples in commercially harvested Dungeness crab during 1993-1994 from various Alaskan fisheries management areas [25]. Data source: Alaska Department of Environmental Conservation – Environmental Health Laboratory.

Sampling	Total number	Total number	Percent
region	crab tested	crab tested	crab tested
		> 80 µg STX eq.	> 80 µg STX eq.
		100 g viscera^-1^	100 g viscera^-1^
Kodiak	391	119	30.4%
Southeast	867	13	1.5%
Yakutat	45	0	0.0%
Prince William Sound	54	0	0.0%
Cook Inlet	101	0	0.0%
**TOTAL**	**1,458**	**132**	**9.1%**

### 3.2. Commercially harvested Alaskan Dungeness crab ex-vessel landings and values

**Fig 5:**
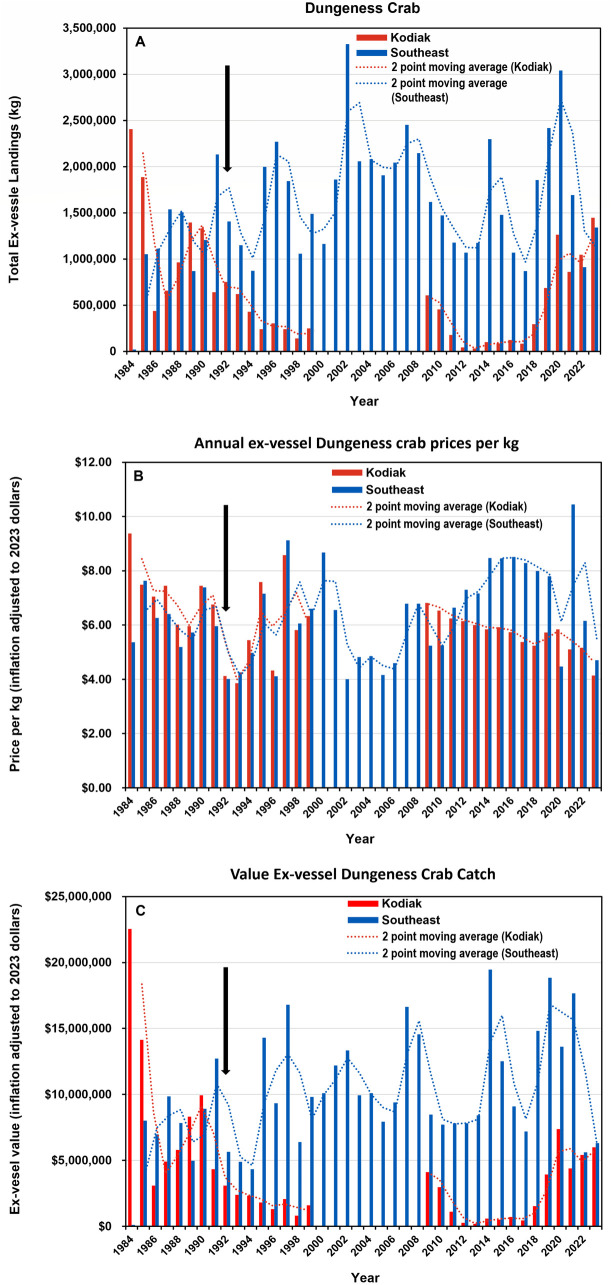
Annual Dungeness crab ex-vessel landings, inflation adjusted prices and harvest value. (A) Annual ex-vessel harvest from Southeast Alaska and Kodiak management areas from 1984 to 2023 in kilograms. (B) Average annual prices in 2023 inflation adjusted US dollars per kilogram. (C) The total value of the annual ex-vessel landing in 2023 inflation adjusted to 2023 US dollars. The dotted lines in each panel represent two-point running averages. The black arrow in each panel indicates 1992, the first year Dungeness crab from the Kodiak fishery management area were required to be eviscerated prior to marketing. Data source: Alaska Department of Fish and Game.

Ex-vessel landings of commercially harvested Dungeness crab from the Kodiak and the SE AK management areas between 1984 and 2023 were highly variable and did not covary (Fig 5A). There was a pronounced decline in Kodiak landings from 1990 to 1999. This decline began before the regulation requiring evisceration of crab from the Kodiak management area was implemented. Between 2000 and 2008 landings data were not available from ADF&G for Kodiak because of low participation in the fishery (see section 2.4). In recent years there has been a revival of the Kodiak Dungeness fishery. This started with the landings from 2018 through 2023 with a peak in 2020 (1.26 million kg; Fig 5). From 1984 to 2023, landings in SE AK varied from 19,232 kg in 1984 to 3.33 million kg in 2002. The average harvest was more than 1.6 million kg per year (std. dev. 649,564 kg) and it exceeded three million kg in both 2002 and 2020 (Fig 5).

Between 1984 and 1999, the ex-vessel price kg^-1^ for crab caught in the Kodiak and SE AK management areas corrected for inflation was slightly higher in Kodiak than SE AK (Fig 5B; average $6.47 ± $1.56 in Kodiak; $6.01 ± 1.39 in SE AK). During this 15-year period prices per kg^-1^ in both regions covaried. From 2013 to 2023 prices were consistently higher in the SE AK management area (average $5.46 ± $0.54 Kodiak; $7.501 ± $1.77 SE AK) except for 2020 when prices were higher in Kodiak. The relatively stable price kg^-1^ for both regions

occurred despite large fluctuations in landings indicating that ex-vessel prices were not impacted by variations in catch (Fig 5B,C; S2 Fig). After 1991, the harvests from Kodiak have been lower than from SE AK except in 2022 when they were nearly equal (Fig 5C). The net result of differences in total harvest and price kg^-1^ has led to a lower annual value of ex-vessel harvest from Kodiak compared to SE AK since 1991 (Fig 5C). The only exception was 2022 when a major drop in SE AK landings resulted in near equal harvest values in the two regions.

### 3.3. Changes in processed Dungeness crab products and values from 1984 through 2023

**Fig 6:**
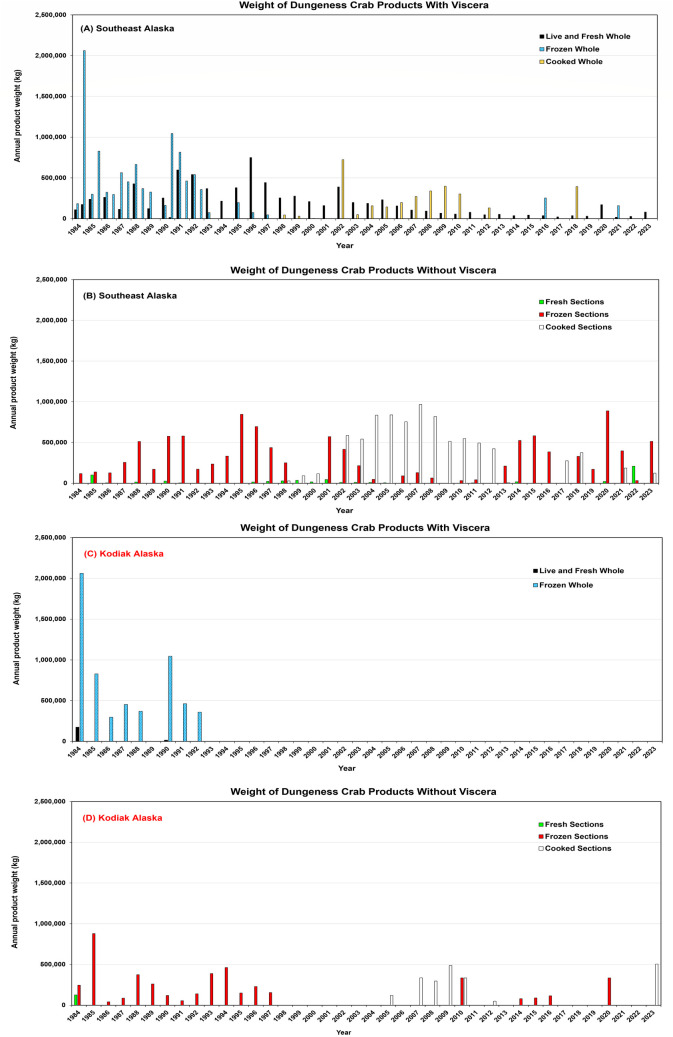
Annual weight of processed Alaskan Dungeness crab products from 1984 to 2001. (A) Kilograms of live, fresh whole, frozen whole and cooked whole crab products processed in the Southeast Alaska management area by year which contained viscera. (B) Kilograms of fresh, frozen and cooked sections processed in the Southeast Alaska management area by year which did not contain viscera. (C) Kilograms of live, fresh whole, frozen whole and cooked whole crab products processed in the Kodiak management area by year which contained viscera. (D) Kilograms of fresh, frozen and cooked sections processed in the Kodiak management area by year that did not contain viscera. Data source: Alaska Department of Fish and Game.

Data on processed Dungeness crab products from SE AK and Kodiak were categorized based on whether they contained viscera (live and fresh, frozen, or cooked whole crab) or did not contain viscera (fresh, frozen and cooked sections) (Fig 6, S3 and S4 Figs). Although variable from year to year, the overall production (kg) of processed products containing viscera gradually declined in SE AK after 1992 (Fig 6A). This occurred even though evisceration was not required for the SE AK management area. During the same time, production of products without viscera increased (Fig 6B). In Kodiak, production of viscera containing products ceased after 1992 (Fig 6C). Products without viscera continued to be produced, though with significant inter annual variability (Fig 6D). There was no obvious up or down trend in the amounts of product being produced. The value of the various products with and without viscera followed a similar pattern for SE AK and Kodiak respectively (S3 Fig). Products without viscera accounted for the major portion of revenue in SE AK, though during a few years cooked or frozen whole crab accounted for a substantial portion of total revenue. In Kodiak after 1992, the only revenue was from the sale of processed products lacking viscera. The price per kg data showed substantial interannual variation but generally fell in the $10 to $25 kg^-1^ range (S4 Fig). There was not as much difference over the past decade in processed product prices between SE AK and Kodiak as observed for the ex-vessel prices (Figs 5,6).

### 3.4. Saxitoxin concentrations in Alaskan Tanner and King crab

**Fig 7:**
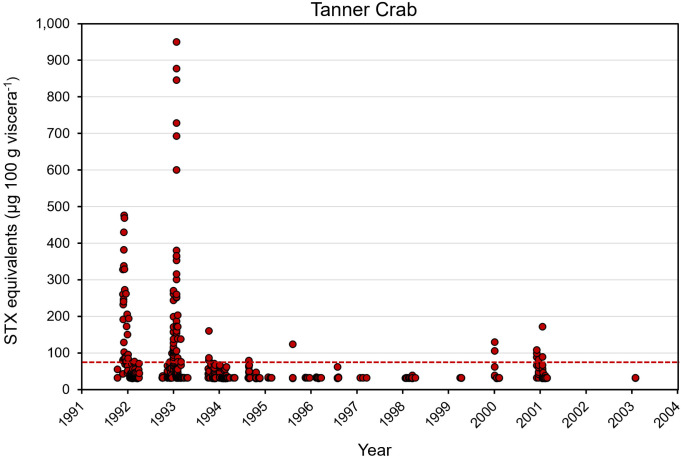
Saxitoxin concentrations in the viscera of Tanner crab (*Chionocetes tanner, C. opilio, C. baridi*) sampled between 1992 and 2004. Data are included from all Alaskan commercial fishery management areas, N = 726. The red line indicates the 80 µg STX eq. 100 g viscera^-1^ regulatory limit for safe harvest. Data source: Alaska Department of Environmental Conservation – Environmental Health Laboratory.

**Fig 8:**
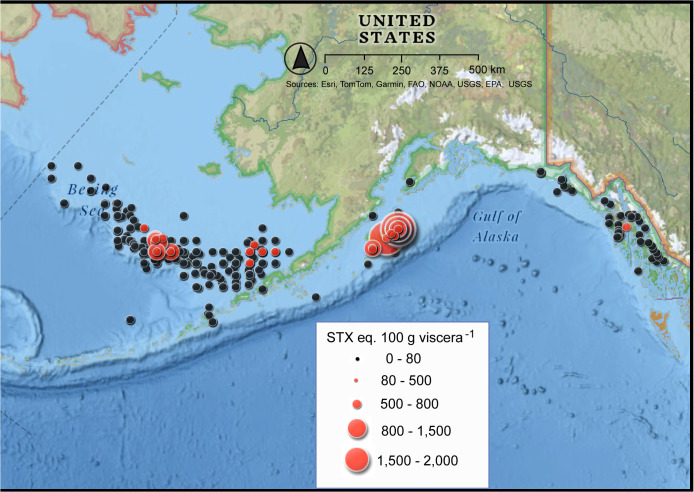
Geographic distribution of saxitoxin concentrations in the viscera of commercially harvested Alaska Tanner (Snow) crab. Data included all available information from fishery management areas between 1992 and 2004. The size of the dot representing each sample is proportional to the toxin level. N = 726. Data source Alaska Department of Environmental Conservation – Environmental Health Laboratory. The toxin concentrations were plotted on Esri’s National Geographic Base Map – Sources: National Geographic, Esri, DeLorme, HERE, UNEP-WCMC, USGS, NASA, ESA, METI, NRCAN, GEBCO, NOAA, iPC.

**Fig 9:**
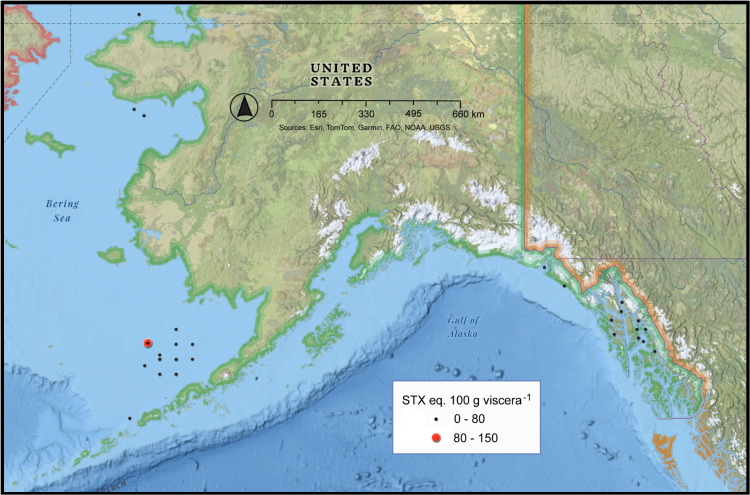
Geographic distribution of saxitoxin concentrations in the viscera of commercially harvested King crab. The data include samples from all fishery management areas collected from 1992 to 2004. N = 40. Data source Alaska Department of Environmental Conservation – Environmental Health Laboratory. The toxin concentrations were plotted on Esri’s National Geographic Base Map – Sources: National Geographic, Esri, DeLorme, HERE, UNEP-WCMC, USGS, NASA, ESA, METI, NRCAN, GEBCO, NOAA, iPC.

Tanner and King crab are typically harvested in deeper waters compared to Dungeness crab. The time series of crab toxin levels confirmed the viscera of Tanner crab collected between 1993 and 2005 exceeded the regulatory limit in 3.7% of the samples (Fig 7; Table 2). Geographically, elevated viscera STX levels were only found in Tanner crab landed near Kodiak or from St. Paul and St. George Islands in the Bering Sea (Fig 8). Sampling of King crab has been much less frequent than for Dungeness or Tanner crab. However, between 1992 and 2001, there was one sample containing 120 µg STX eq. 100 g viscera^-1^ that exceeded regulatory limits (Fig 9, S5 Fig). This King crab was collected in the Bering Sea about halfway between Port Heiden and St. Paul Island at a depth of 130 m in November 1993. All other 39 King crab tested below the regulatory limit for STX.

**Table 2:** Saxitoxin concentrations in commercially harvested Alaskan Tanner crab (*Chionocetes tanneri, C. opilio, C. baridi*) sampled between 1992-2004. The data were categorized based on levels ranging from 0-80 µg STX eq. 100 g viscera^-1^ (advisory safety limit) to > 800 µg STX eq 100 g viscera^-1^. N = 726. Data source: Alaska Department of Environmental Conservation – Environmental Health Laboratory.

STX eq.	Total number	Percent of total
(µg 100 g viscera^-1^)	Tanner crab	Tanner crab
	tested	tested
0-80	648	89.3%
80-250	51	7.0%
250-500	21	2.9%
500-800	3	0.4%
800-900	3	0.4%
Total	726	100%

### 3.5. Regional differences in *Alexandrium catenella* cell toxicities

**Fig 10:**
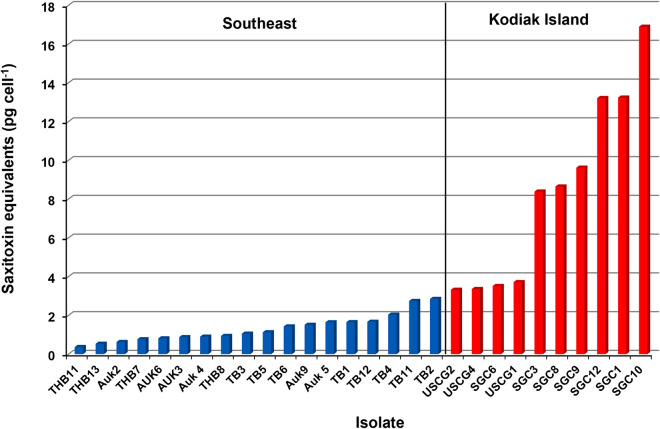
Saxitoxin concentrations per cell of *Alexandrium catenella* isolates from Kodiak and Southeast Alaska. Isolate designations starting with TB or TBH were collected from Thomas Basin, Ketchikan, AK (55.33952, −131.642501). Those starting with Auk were isolated from Auke Bay near Juneau, AK (58.384143, −134.649619) and those starting with SGC or USCG were collected near the U.S. Coast Guard Station, Kodiak, AK (57.743296, −152.484128). Blue bars indicate isolates obtained from the SE AK management area and the red bars are those from the Kodiak management area.

One possibility for the differences in toxicity between the Kodiak and SE AK management areas could be due to regional differences in the STX content of *A. catenella* cells. The toxicity of 10 isolates from the Kodiak management area (x̄ = 8.39, SD = 4.91) were compared to those of 19 isolates from SE AK management area (x̄ = 1.31, SD = 0.71). The per cell toxicities of the Kodiak isolates were significantly higher than those from the SE AK region, t = −6.31, p = < .00001 (Fig 10). These regional variations in STX per cell correspond with the differences in the frequency of crab containing above regulatory STX concentrations (Fig 2).

## 4. Discussion

An assessment of historical ADEC-EHL data confirms that STX levels in the Kodiak fishery were higher and more frequent than in other Alaskan harvest areas. The likelihood of harvesting toxic Dungeness crab from Kodiak was 60.5% (1992–2023, N = 2,080, Fig 2B) versus 8.1% (1994–2023, N = 940, Figs 2C,4) in SE AK. Starting in 1992, after Dungeness crab tested positive for STX, ADF&G restricted the sale of live or whole cooked crab from the Kodiak area. Although the incidence of toxicity in the SE AK Dungeness crab fishery was low, crab with toxin levels above the STX regulatory limit were also detected in this region. Based on the available data, this toxicity was most pronounced in the Haines region though other locations in SE AK were implicated (Fig 4). The presence of high STX concentrations in crab from SE AK suggestes additional monitoring is warranted in sentinel locations, particularly given climate change related increases in water temperature that may promote the development of toxic *A. catenella* blooms [26–29].

When evaluating the Alaskan Dungeness crab fishery, it is important to understand how it has evolved. In the 1960s, driven by high market demand resulting from low harvests in California and the Pacific Northwest (PNW – WA and OR coast), a few large vessels were able to harvest an average of one million kg of Dungeness crab per year [30]. However, by the 1970s production in the PNW rebounded and demand for Alaskan crab declined. During this 1970s period there was limited infrastructure to support the early Alaskan fishery, with few processors available to handle the catch. Fishermen either had to sell their catch at the dock or ship live crab by airfreight. With only 31 permit holders and a fleet of small vessels, the average annual catches were around 277,000 kg. Participation was low and the year-round fishery initially had minimal regulation [30]. In the mid-1970s crab harvesting regulations were put in place to prevent the by-catch of female Dungeness and Red King crab [31].

Between the 1981–1982 and 1991–1992 harvest seasons, the industry underwent extensive change. Dungeness crab harvests in Pacific coast states once again declined and processors responded by purchasing more crab from AK [32]. Concurrently, the fishery transitioned from a small, traditional group of single species participants to a secondary fishery with a larger number of new, transitory fishermen. On average 173 permit holders per year in SE AK landed 1.1 million kg of Dungeness crab. By 1991, the greatly increased participation in the fishery and the potential to overharvest available stocks led to a four-year moratorium on new permits. In 1996 the moratorium was lifted and a tiered permit system based on the number of permit holders and total number of crab pots was instituted [14].

By the 2000s, the fishery reached its current form with approximately 180–275 annual permit holders. Ex-vessel landings of Dungeness crab were cyclic [33], but did not covary between the two management regions (Fig 5A). The lack of covariance is not unexpected given the myriad factors, both biotic and abiotic, that affected the harvest and prices. Many of these factors were detailed in ADF&G’s Fishery Management Reports and included variations in crab recruitment, numbers of vessel permits and processors handling Dungeness crab, fishing effort, length of harvest season, and the evisceration requirement for commercially harvested Kodiak Dungeness crab [14,15,32,34,35].

These various factors were likely responsible for the divergence in the trajectory of the Kodiak and SE AK Dungeness crab fisheries after 1990. Specifically, Kodiak landings declined from 1990 to 1998 (Fig 5A) with participation decreasing from 64 vessels in 1990–11 in 1998 [36]. This decline was likely due to a recruitment failure that reduced Dungeness crab population size, and only secondarily to the 1992 ADF&G Kodiak evisceration requirement [36]. This conclusion is supported by the decline in landings that started before the implementation of the evisceration regulation (Fig 5A). The missing data from 2000 to 2008 do not allow for a continuous record of the Kodiak landings but suggest that fewer than three vessels or processors were operating in Kodiak during that time. Given the small number of boats operating in the area, harvests would have been low. In contrast, after declining from 1991 to 1994, landings from SE AK ranged between 1–3 million kg annually (Fig 5A).

The ex-vessel Dungeness crab prices kg^-1^ received in Kodiak versus SE AK between 1984 and 2023 showed two distinctly different patterns (S2 Fig). Plots comparing landings versus price kg^-1^ indicated that catch size did not impact Dungeness crab ex-vessel price with one notable exception for SE AK’s bountiful harvest in 2020 (Fig 5). Between 1984 and 1999, as the fishery was maturing and regulations were being established, there was little difference in price per kg between Kodiak and SE (Fig 5B). This similarity in prices persisted despite the discovery of STX in crab viscera in the early 1990s. The total ex-vessel value of the SE AK Dungeness crab fishery exceeded that of Kodiak in five of the nine years before 1992 (Fig 5C). After 1992 the values of the SE AK landings substantially exceeded those from Kodiak. These data did not provide clear insight into whether the evisceration requirement for Kodiak influenced the ex-vessel prices received after 1992. From 2009 to 2023, ex-vessel prices kg^-1^ generally declined in Kodiak. In SE AK, prices kg^-1^ were more variable but remained on average approximately $2.00 kg^-1^ higher than those received in Kodiak. Additionally, prices in Kodiak were lower compared to other locations on the U.S. west coast [36]. Transportation costs from Kodiak may have contributed to this difference.

This study also examined the wholesale value of various processed Dungeness crab products (Fig 6, S3 and S4 Figs). Prior to 1992, live, fresh and frozen whole crab containing viscera dominated processed products in both SE AK and Kodiak (Fig 6C, D). Following the implementation of the 1992 evisceration regulation only fresh, frozen and cooked sections without viscera were produced in Kodiak (Fig 6C, D; S3 Fig). Though substantial quantities of whole crab products containing viscera were still produced in SE AK, there was a shift toward declining production of whole crab products containing viscera and steady or slightly increased production of processed products without viscera. This shift toward fresh, frozen and cooked sections occurred although there was no requirement to eviscerate. The data also confirmed the dominance of the SE AK versus the Kodiak fishery after 1990 in terms of the total amount (kg) of processed products. This shift is likely due to environmental or other factors causing a decline in the Kodiak stock and only perhaps secondarily to the evisceration requirement [36]. The price kg^-1^ data showed the processed products without viscera were more valuable than the whole crab products containing viscera (S4 Fig). Overall, these data were consistent with a shift in the wholesale industry toward producing higher value Dungeness crab products associated with lower PSP risk.

Toxin levels in other commercial crab species from all Alaskan management areas were also examined. Only 10.7% of the 726 Tanner (Snow) crab tested above the regulatory limit of 80 µg STX 100 g viscera^-1^ between 1992 and 2004. However, it is important to note that in some samples, STX concentrations surpassed 800 µg STX 100 g viscera^-1^ (Table 2, Fig 7). The distribution map for Tanner crab indicated few toxic Tanner crab were found in the SE AK area. The Tanner crab with the highest PST levels were found in the Kodiak management area with some PST-contaminated Tanner crab from the Saint Paul and Saint George Islands along the Bering Sea shelf (Fig 8). Given that the most recent testing of Tanner crab was conducted in 2004, and *A. catenella* blooms are of concern in the Arctic due to increasing ocean temperatures [27,37,38], monitoring for STX in the Bering Sea should be reviewed [28].

While toxicity records are available for only 40 King crab samples, these few data are valuable (Fig 9; S5 Fig). They include crab samples from a wide geographic area including Kotzebue Sound (Chukchi Sea), Norton Sound (Bering Sea) and the Bering Sea Shelf off Bristol Bay. The only King crab sample positive for STX was collected on the Bering Sea Shelf, about mid-way between the Pribilof Islands and Port Heiden on the north shore of the Alaskan Peninsula. Since commercial King crab fishing is a winter activity, and not in synchrony with the bloom period of *A. catenella* (May-Aug, [27]), it was surprising to discover even a single King crab with detectable STX. In contrast, subsistence (personal use) King crab harvest often occurred during the summer. Consequently, understanding that STX may vector into King crab could be of concern if *A. catenella* blooms in Arctic areas become more frequent.

It is of note that the toxicity patterns observed in the Kodiak and the Pribilof Islands were consistent with a pattern of *A. catenella* blooms in the Gulf of Alaska where they were most abundant in fjords and nearshore regions (Figs 5,9,10). The viscera of Tanner and King crab collected farther from land remained below the regulatory STX value. This finding was congruent with data from Lower Cook Inlet which showed high density blooms of *A. catenella* originate and thrive in shallow nearshore areas and bays receiving sufficient runoff to produce stable vertical stratification with a mesohaline surface layer [27,39–41]. Data presented by Vandersea et al. [27] show bloom populations can be exported to the surrounding offshore regions where STX may vector to benthic invertebrates. The bays are also shallow enough to retain viable *Alexandrium* cyst beds that contribute to blooms in subsequent years. The data from Kodiak and the Haines regions indicate there are “hotspots” where *A. catenella* blooms tend to occur more frequently and are more intense. A major question is if regular monitoring of a relatively few sentinel “hot spots” could provide a reliable early warning of when blooms are likely to develop and vector into shellfish and crab populations.

Another finding that could prove valuable in explaining why the Dungeness crab in the Kodiak management area are more toxic compared to those in SE AK comes from preliminary data on the toxicity of *A. catenella* cell isolates from these two regions. *Alexandrium catenella* cells from SE AK produced less STX, on average, than those from Kodiak (Fig 10). This confirms earlier work reported by Hall [42] showing that *A. catenella* isolates from different populations in Alaska produced different amounts and types of STX congeners. Longitudinal differences in *Alexandrium* toxicity have also been observed in U.S. east coast populations [43]. It is important to note the method used to generate these data (Abraxis ELISA STX test kit) primarily detects STX and is less sensitive to other congeners of STX like neosaxitoxin. Some congeners like neosaxitoxin have equivalent or greater toxicity than STX [44]. If local *A. catenella* populations (e.g. those in SE AK) predominately produce neosaxitoxin or other congeners with toxicity similar to saxitoxin but are not detected by the ELISA method, overall toxicity will be underestimated. Therefore, the ELISA results do not definitively demonstrate that *Alexandrium* populations in Kodiak contained more STX than in other areas. Consequently, investigating the toxicity differences in regional *A. catenella* populations using high resolution detection methods (LCMS) which quantify STX congeners should be a high research priority for management purposes.

## Conclusions

A summary of the results from this study supports the following conclusions:

- Findings concur with ADF&G’s decision to require evisceration of commercially harvested Dungeness crab from the Kodiak management area for safe consumption.
- Implementation of the 1992 evisceration requirement was associated with a shift toward sales of higher value, fresh, cooked and frozen sections and away from whole crab products containing viscera.
- Identification of sentinel locations or “hot spots” for seasonal monitoring of *A. catenella* blooms is recommended to inform management decisions for safe harvesting of AK crab.
- Evidence supports the Haines area of SE AK is a hot spot where site-specific monitoring for STX may be needed.
- Factors governing Dungeness crab harvests in Kodiak and SE AK do not co-vary due to uncoupled decadal changes in the numbers of vessel permits, processors, effort and crab population levels.
- STX values above regulatory levels are observed in a low percentage of Tanner crab, indicating additional sampling may be warranted to assess current toxicity levels found in viscera.
- Preliminary evidence on toxicity differences in *A. catenella* strains from different geographic regions may account for variations in STX concentrations observed in Dungeness crab from Kodiak and SE AK.

## Supporting information

S1 Fig Alaska Department of Fish and Game southeast Alaska shellfish statistical harvest area map -https://www.adfg.alaska.gov/static/fishing/PDFs/commercial/chart05_seak_gf.pdf. This map illustrates how shellfish harvest statistical areas are divided into defined polygons. Because the Dungeness, King and Tanner crab have different geographic distributions, the regional statistical area maps used for collecting species-specific harvest data vary. In this study, the harvest location of the Dungeness, Tanner and King crab samples analyzed for STX were designated by the polygon identification number where they were collected. This map is republished from Alaska Department of Fish and Game under a CC BY license with permission of the Alaska Department of Fish and Game 2011. Depending on the type of crab being harvested, the relevant polygon identification number was obtained from one of the various shellfish regional maps listed below. To plot the STX data on maps, samples were assigned the center latitude and longitude of the polygon where they were collected using ADF&G maps. In some cases, samples were harvested from two or more contiguous areas before they were landed. In these instances, the latitude and longitude assigned to each sample was the approximate center point of the adjacent polygons. https://www.adfg.alaska.gov/static/fishing/PDFs/commercial/chart01_gulf.pdf, https://www.adfg.alaska.gov/static/fishing/PDFs/commercial/chart02_akpen_ai.pdf, https://www.adfg.alaska.gov/static/fishing/PDFs/commercial/chart03_bs.pdf, https://www.adfg.alaska.gov/static/fishing/PDFs/commercial/chart04_nbs.pdf, https://www.adfg.alaska.gov/static/fishing/PDFs/commercial/maps/chart04_king_tanner_r1_all.pdf, https://www.adfg.alaska.gov/static/fishing/PDFs/commercial/maps/chart05a_salm_shell_juneau.pdf, https://www.adfg.alaska.gov/static/fishing/PDFs/commercial/maps/chart05b_salm_shell_ketchikan.pdf, https://www.adfg.alaska.gov/static/fishing/PDFs/commercial/maps/chart05c_salm_shell_petersburg.pdf, https://www.adfg.alaska.gov/static/fishing/PDFs/commercial/maps/chart05d_salm_shell_sitka.pdf, https://www.adfg.alaska.gov/static/fishing/PDFs/commercial/chart08_kodiak.pdf, https//www.adfg.alaska.gov/static/fishing/PDFs/commercial/chart09_akpenn_chignik.pdf, https://www.adfg.alaska.gov/static/fishing/PDFs/commercial/chart10_ai.pdf
(TIFF)

S2 Fig Annual commercially harvested Dungeness crab landings (kg) versus their total value in 2023 inflation-adjusted U.S. dollars. (A) Kodiak. (B) Southeast Alaska. Data source Alaska Department of Fish and Game.
(TIFF)

S3 Fig Annual value of processed Alaskan Dungeness crab products from 1984 to 2023. (A) The annual value of live and fresh whole, frozen whole and cooked whole crab products containing viscera processed in Southeast Alaska management area in 2023 inflation-adjusted dollars. (B) The annual value of live and fresh whole, frozen whole and cooked whole crab products without viscera processed in the Southeast Alaska management area in 2023 inflation-adjusted dollars. (C) The annual value of live and fresh whole, frozen whole and cooked whole crab products containing viscera processed in the Kodiak Alaska management area in 2023 inflation-adjusted dollars. (D) The annual value of live and fresh whole, frozen whole and cooked whole crab products without viscera processed in the Kodiak Alaska management area in 2023 inflation-adjusted dollars. Data source: Alaska Department of Fish and Game.
(TIFF)

S4 Fig Price per kilogram of various commercially harvested Alaskan Dungeness crab from 1984 to 2023. (A) The annual price kg^-1^ of live and fresh whole, frozen whole and cooked whole crab products containing viscera processed in the Southeast Alaska management area in 2023 inflation-adjusted dollars. (B) The annual price kg^-1^ of live and fresh whole, frozen whole and cooked whole crab products without viscera processed in the Southeast Alaska management area in 2023 inflation-adjusted dollars (C) The annual price kg^-1^ of live and fresh whole, frozen whole and cooked whole crab products containing viscera processed in the Kodiak management area in 2023 inflation-adjusted dollars. (D) The annual price kg^-1^ of live and fresh whole, frozen whole and cooked whole crab products without viscera processed in the Kodiak management area in 2023 inflation adjusted dollars. Data source: Alaska Department of Fish and Game.
(TIFF)

S5 Fig Time series of saxitoxin (STX) concentrations in the viscera of commercially harvested Alaska King crab. Samples were collected between 1992 and 2001. N = 40. The red line indicates the regulatory limit of 80 µg STX eq. 100 g viscera^-1^ for safe harvest. Data source: Alaska Department of Environmental Conservation – Environmental Health Laboratory.
(TIFF)

## References

[1] Stromme G. PSP and the Alaska crab fishery. In: RaLonde R, Painter R, editors. Living with paralytic shellfish poisoning: A conference to develop PSP research and management strategies for safe utilization of shellfish in Alaska. Fairbanks, Alaska: Alaska Sea Grant Program; 1995.

[2] Wekell MM. Seafood Toxins. J Assoc Anal Chem. 1991;74(1):137–41. doi: 10.1093/jaoac/74.1.137

[3] McLaughlin J. Paralytic Shellfish Poisoning — Alaska, 1993–2014: State of Alaska Epidemiology Bulletin; 2015 [cited 2025 Apr 3]. Available from: https://epi.alaska.gov/bulletins/docs/b2015_01.pdf

[4] Bennett C, Stryker K. Recent Alaska death due to paralytic shellfish poisoning; Alaskans should know the health risks when harvesting shellfish State of Alaska Department of Health and Social Services; 2020 [cited 2025 Apr 3]. Available from: https://aoos.org/wp-content/uploads/2019/06/DHSS_PressRelease_PSPFatality_20200715.pdf

[5] HWSR. Shellfish Safety. Report 5. Washington State Department of Health. 2012.

[6] Gordon E, Fogtmann D. Fishery Notice - Fisheries and Oceans Canada, FN0486-PSP. Bivalve Shellfish Marine Biotoxin Advisory. Widespread closures on the B.C. Coast due to PSP 2010. 2010 [cited 2024 May 9]. Available from: https://sportfishingbc.com/threads/pay-attention-to-this-notice.43658/

[7] Esri. Esri National Geographic Base Map. 2025 [cited 2025 May 12]. Available from: https://www.arcgis.com/home/item.html?id=f33a34de3a294590ab48f246e99958c9

[8] ISSCNSSP. National Shellfish Sanitation Program (NSSP). Guide for the control of molluscan shellfish 2019 revision. 2020 [cited 2024 Mar 8]. Available from: https://www.fda.gov/media/143238/download?attachment

[9] Shumway SE. Phycotoxin‐related shellfish poisoning: Bivalve molluscs are not the only vectors. Rev Fisher Sci. 1995;3(1):1–31. doi: 10.1080/10641269509388565

[10] Deeds JR, Landsberg JH, Etheridge SM, Pitcher GC, Longan SW. Non-traditional vectors for paralytic shellfish poisoning. Mar Drugs. 2008;6(2):308–48. doi: 10.3390/md20080015 18728730 PMC2525492

[11] Turner AD, Hatfield RG, Maskrey BH, Algoet M, Lawrence JF. Evaluation of the new European Union reference method for paralytic shellfish toxins in shellfish: a review of twelve years regulatory monitoring using pre-column oxidation LC-FLD. TrAC Trends Anal Chem. 2019;113:124–39. doi: 10.1016/j.trac.2019.02.005

[12] Wekell JC, Hurst J, Lefebvre K. The origin of the regulatory limits for PSP and ASP toxins in shellfish. J Shellfish Res. 2004;23:927–30. https://www.researchgate.net/publication/285809374_The_origin_of_the_regulatory_limits_for_PSP_and_ASP_toxins_in_shellfish

[13] ESRI. Environmental Systems Research Institute, Inc. 2024 [cited 2024 May 9]. Available from: https://www.esri.com/en-us/about/about-esri/technology

[14] Bergman T, Stratman J, Messmer A, Rebert A, Palof K. Management report for the southeast and Yakutat Dungeness crab fisheries, 2017/18-2019/20. Anchorage, Alaska: Alaska Department of Fish and Game Division of Sport Fish, Research and Technical Services. 2021. pp. 1–40. https://www.adfg.alaska.gov/static/regulations/regprocess/fisheriesboard/pdfs/2021-2022/se/FMR21-25.pdf

[15] Bevaart K, Phillips K. Annual management report for shellfish fisheries in the Kodiak, Chignik, and South Peninsula Districts, 2020. Fishery Management Report No. 21–29. Alaska Department of Fish and Game, Divisions of Sport Fish and Commercial Fisheries. 2021. pp. 1–48. https://www.adfg.alaska.gov/static/regulations/regprocess/fisheriesboard/pdfs/2021-2022/state/fmr21_29.pdf

[16] ADF&FDCFCEV. Commercial Shellfish Fisheries, Registration Area A, Dungeness Crab Fishery Catch, Effort, & Value 2024 [cited 2024 Mar 8]. Available from: https://www.adfg.alaska.gov/index.cfm?adfg=commercialbyareasoutheast.shellfish_harvest_dungenessa

[17] USIC. US inflation calculator: CoinNews family of web sites. 2024 [cited 2024 Dec 18]. Available from: https://www.usinflationcalculator.com/about/

[18] AFSC. Confidentiality and data quality protocols for BSAI crab economic data: A discussion and proposal Alaska Fisheries Science Center. Seattle, WA; NOAA: 2007. Avaiable from: http://npfmc.org/wp-content/PDFdocuments/catch_shares/DATA032007.pdf

[19] COAR. Statewide Crab COAR Production, Commercial Operator’s Annual Reports (COAR): Alaksa Department of Fish and Game; 2024. [cited 2024 May 12] Available from: https://www.adfg.alaska.gov/index.cfm?adfg=fishlicense.coar_crabproduction

[20] ADF&GSAC. Alaska Department of Fish and Game. Information by fishery: Statistical area charts. 2024 [cited 2024 Mar 8]. Available from: https://www.adfg.alaska.gov/index.cfm?adfg=fishingCommercialByFishery.statmaps

[21] News.com P. Alaska Department of Environmental Conservation Seafood August 2, 2011. Southeast Alaska Dungeness crabs show high PSP toxin levels. 2024 [cited 2024 Mar 8]. Available from: https://www.perishablenews.com/seafood/southeast-alaska-dungeness-crabs-show-high-psp-toxin-levels/

[22] Kramer D, Herter H, Stoner A. Handling of fresh crabs and crabmeat. Seagram, Alaska Sea Grant, ASG-48, 1-8. 2009 [cited 2024 Mar 3]. Available from: https://seagrant.uaf.edu/bookstore/pubs/ASG-48.html

[23] Vandersea MW, Kibler SR, Van Sant SB, Tester PA, Sullivan K, Eckert G, et al. qPCR assays for *Alexandrium fundyense* and *A. ostenfeldii* (Dinophyceae) identified from Alaskan waters and a review of species-specific Alexandrium molecular assays. Phycologia. 2017;56(3):303–20. doi: 10.2216/16-41.1 32831405 PMC7441911

[24] Statististics SS. T-Test Calculator for 2 Independent Means 2024 [cited 2024 Dec 18]. Available from: https://www.socscistatistics.com/tests/studentttest/default2.aspx

[25] Wright BA, Dona E, RaLonde R. Environment Alaska, PSP in Dungeness crab in Alaska 2014 [cited 2024 Mar 3]. Available from: https://environmentalaska.us/psp-in-dungeness-crab.html

[26] Gobler CJ, Doherty OM, Hattenrath-Lehmann TK, Griffith AW, Kang Y, Litaker RW. Ocean warming since 1982 has expanded the niche of toxic algal blooms in the North Atlantic and North Pacific oceans. Proc Natl Acad Sci U S A. 2017;114(19):4975–80. doi: 10.1073/pnas.1619575114 28439007 PMC5441705

[27] Vandersea MW, Kibler SR, Tester PA, Holderied K, Hondolero DE, Powell K, et al. Environmental factors influencing the distribution and abundance of Alexandrium catenella in Kachemak bay and lower cook inlet, Alaska. Harmful Algae. 2018;77:81–92. doi: 10.1016/j.hal.2018.06.008 30005804

[28] Anderson DM, Fachon E, Pickart RS, Lin P, Fischer AD, Richlen ML, et al. Evidence for massive and recurrent toxic blooms of Alexandrium catenella in the Alaskan Arctic. Proc Natl Acad Sci U S A. 2021;118(41):e2107387118. doi: 10.1073/pnas.2107387118 34607950 PMC8521661

[29] Ross ARS, Ip B, Mueller M, Surridge B, Hartmann H, Hundal N, et al. Seasonal monitoring of dissolved and particulate algal biotoxins in the northern Salish Sea using high performance liquid chromatography and tandem mass spectrometry. Harmful Algae. 2025;145:102854. doi: 10.1016/j.hal.2025.102854 40324864

[30] Messmer A, Olson A, Kelley S, Stratman J, Wood K. Annual management report for the 2013/2014 Southeast Alaska/Yakutat Dungeness crab fisheries: Fishery management report no. 14-52. 2014. [cited 2024 May 12]. Available from: http://irma.nps.gov/DataStore/DownloadFile/579123

[31] Stratman J, Messmer A, Olson A, Wood K, Kelley S. Annual management report for the 2013/2014 Southeast Alaska/Yakutat Dungeness crab Fisheries: Alaska Department of Fish and Game. Fishery management report no. 14-52. 2014. [cited 2024 May 12]. Available from: https://irma.nps.gov/DataStore/DownloadFile/579121

[32] Stratman J, Wood K, Messmer A. Annual management report for the 2016/17 southeast Alaska/Yakutat Dungeness crab fisheries. Fishery Management Report No 17–55. Anchorage, Alaska: Alaska Department of Fish and Game Division of Sport Fish, Research and Technical Services. 2017. pp. 1–35. https://www.adfg.alaska.gov/FedAidPDFs/FMR17-55.pdf

[33] Woodby D, Carlile D, Siddeek S, Funk F, Clark JH, Hulbert L. Commercial Fisheries of Alaska Special Publication No. 05-09. 2005. [cited 2024 May 12] Available from: https://www.adfg.alaska.gov/fedaidpdfs/sp05-09.pdf

[34] Bevaart K. Annual management report for shellfish fisheries in the Kodiak, Chignik, and South Peninsula districts, 2021: Alaska Department of Fish and Game Divisions of Sport Fish and Commercial Fisheries; 2022 [cited 2024 May 12]. Available from: https://www.adfg.alaska.gov/FedAidPDFs/FMR22-18.pdf

[35] Whiteside C, Looman A. Annual management report for shellfish fisheries in the Kodiak, Chignik, and South Peninsula districts, 2022 2023 [cited 2024 May 12]. Available from: https://www.adfg.alaska.gov/FedAidPDFs/FMR23-17.pdf

[36] Ruccio MP, Jackson DR. Kodiak and Alaska Peninsula Commercial Dungeness Crab Fisheries, 1999. Report to the Alaska Board of Fisheries. Regional Information Report No. 4K00-3. 2000 [cited 2024 May 12]. Available from: https://www.adfg.alaska.gov/FedAidPDFs/RIR.4K.2000.03.pdf

[37] Bill BD, Moore SK, Hay LR, Anderson DM, Trainer VL. Effects of temperature and salinity on the growth of Alexandrium (Dinophyceae) isolates from the Salish Sea. J Phycol. 2016;52(2):230–8. doi: 10.1111/jpy.12386 27037588 PMC4818979

[38] Natsuike M, Saito R, Fujiwara A, Matsuno K, Yamaguchi A, Shiga N, et al. Evidence of increased toxic Alexandrium tamarense dinoflagellate blooms in the eastern Bering Sea in the summers of 2004 and 2005. PLoS One. 2017;12(11):e0188565. doi: 10.1371/journal.pone.0188565 29182651 PMC5705126

[39] Neal RA. Fluctuations in the levels of paralytic shellfish toxin in four species of lamellibranch mollusks near Ketchikan, Alaska, 1963-1965. Dissertation. University of Washington; Seattle: 1967. pp. 149.

[40] Smayda TJ. Harmful algal blooms: Their ecophysiology and general relevance to phytoplankton blooms in the sea. Limnol Oceanogr. 1997;42(5part2):1137–53. doi: 10.4319/lo.1997.42.5_part_2.1137

[41] Ryan J, Dierssen H, Kudela R, Scholin C, Johnson K, Sullivan J, et al. Coastal Ocean Physics and Red Tides: An Example from Monterey Bay, California. oceanog. 2005;18(2):246–55. doi: 10.5670/oceanog.2005.58

[42] Hall S. Toxins and toxicity of *Protogonyaulax* from the Northeast Pacific. Ph D dissertation. University of Alaska; Fairbanks: 1982. pp. 196. https://scholarworks.alaska.edu/bitstream/handle/11122/9288/Hall_S_1982.pdf?sequence=1

[43] Maranda L, Anderson DM, Shimizu Y. Comparison of toxicity between populations of Gonyaulax tamarensis of eastern North American waters. Estuar Coast Shelf Sci. 1985;21(3):401–10. doi: 10.1016/0272-7714(85)90020-4

[44] Turnbull AR, Harwood DT, Boundy MJ, Holland PT, Hallegraeff G, Malhi N, et al. Paralytic shellfish toxins - Call for uniform reporting units. Toxicon. 2020;178:59–60. doi: 10.1016/j.toxicon.2020.02.018 32250748

