# Supplementary figures and images for: Saxitoxin in Alaskan commercial crab species
Source: PLoS One. 2025 Sep 3;20(9):e0330132. doi: 10.1371/journal.pone.0330132 (PMC12407428; doi:10.1371/journal.pone.0330132)

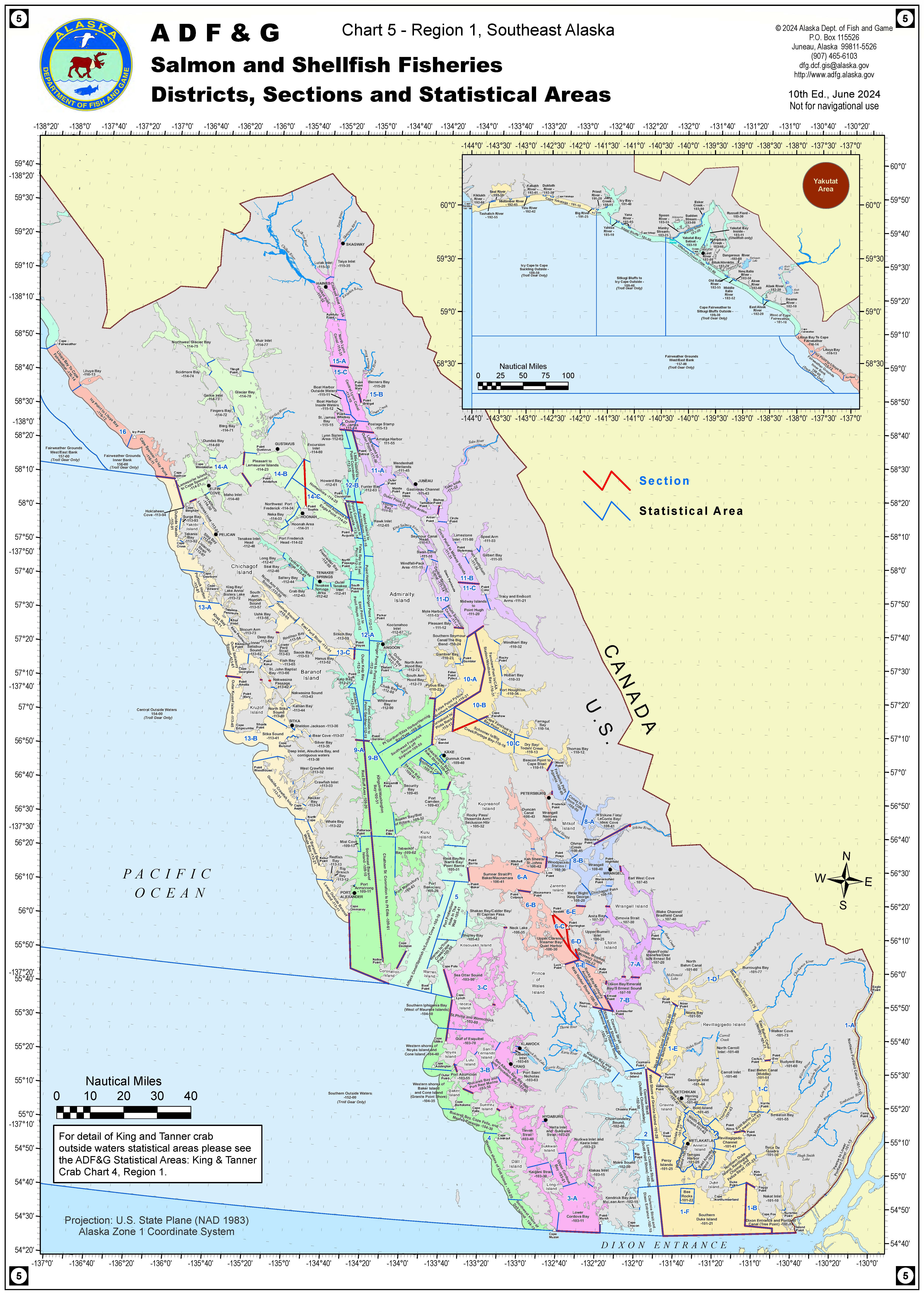

Supplement: S1 Fig — This map illustrates how shellfish harvest statistical areas are divided into defined polygons. Because the Dungeness, King and Tanner crab have different geographic distributions, the regional statistical area maps used for collecting species-specific harvest data vary. In this study, the harvest location of the Dungeness, Tanner and King crab samples analyzed for STX were designated by the polygon identification number where they were collected. This map is republished from Alaska Department of Fish and Game under a CC BY license with permission of the Alaska Department of Fish and Game 2011. Depending on the type of crab being harvested, the relevant polygon identification number was obtained from one of the various shellfish regional maps listed below. To plot the STX data on maps, samples were assigned the center latitude and longitude of the polygon where they were collected using ADF&G maps. In some cases, samples were harvested from two or more contiguous areas before they were landed. In these instances, the latitude and longitude assigned to each sample was the approximate center point of the adjacent polygons. https://www.adfg.alaska.gov/static/fishing/PDFs/commercial/chart01_gulf.pdf, https://www.adfg.alaska.gov/static/fishing/PDFs/commercial/chart02_akpen_ai.pdf, https://www.adfg.alaska.gov/static/fishing/PDFs/commercial/chart03_bs.pdf, https://www.adfg.alaska.gov/static/fishing/PDFs/commercial/chart04_nbs.pdf, https://www.adfg.alaska.gov/static/fishing/PDFs/commercial/maps/chart04_king_tanner_r1_all.pdf, https://www.adfg.alaska.gov/static/fishing/PDFs/commercial/maps/chart05a_salm_shell_juneau.pdf, https://www.adfg.alaska.gov/static/fishing/PDFs/commercial/maps/chart05b_salm_shell_ketchikan.pdf, https://www.adfg.alaska.gov/static/fishing/PDFs/commercial/maps/chart05c_salm_shell_petersburg.pdf, https://www.adfg.alaska.gov/static/fishing/PDFs/commercial/maps/chart05d_salm_shell_sitka.pdf, https://www.adfg.alaska.gov/static/fishing/PDFs/commercial/chart08 [file pone.0330132.s001.tiff]

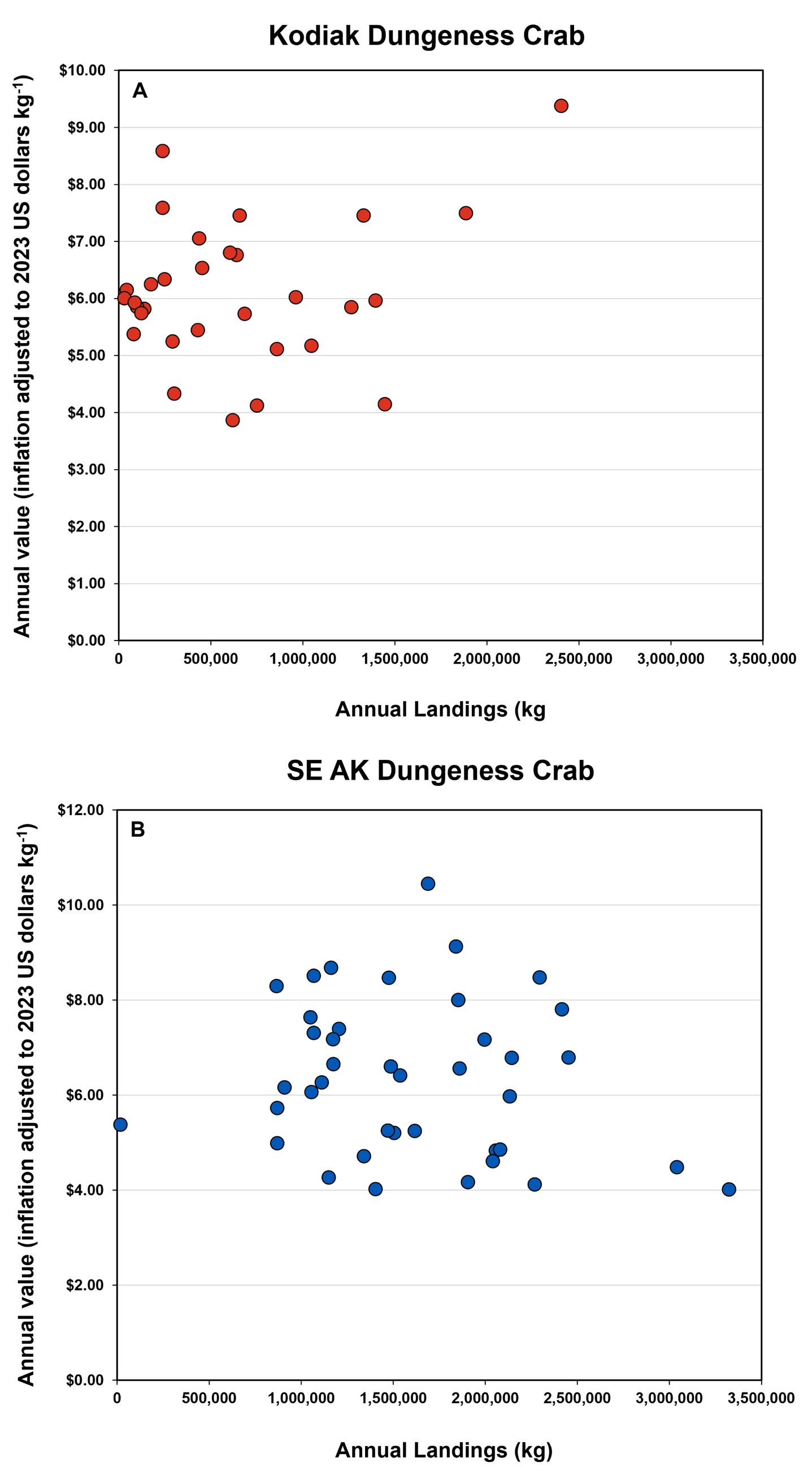

Supplement: S2 Fig — (A) Kodiak. (B) Southeast Alaska. Data source Alaska Department of Fish and Game. (TIFF) [file pone.0330132.s002.tif]

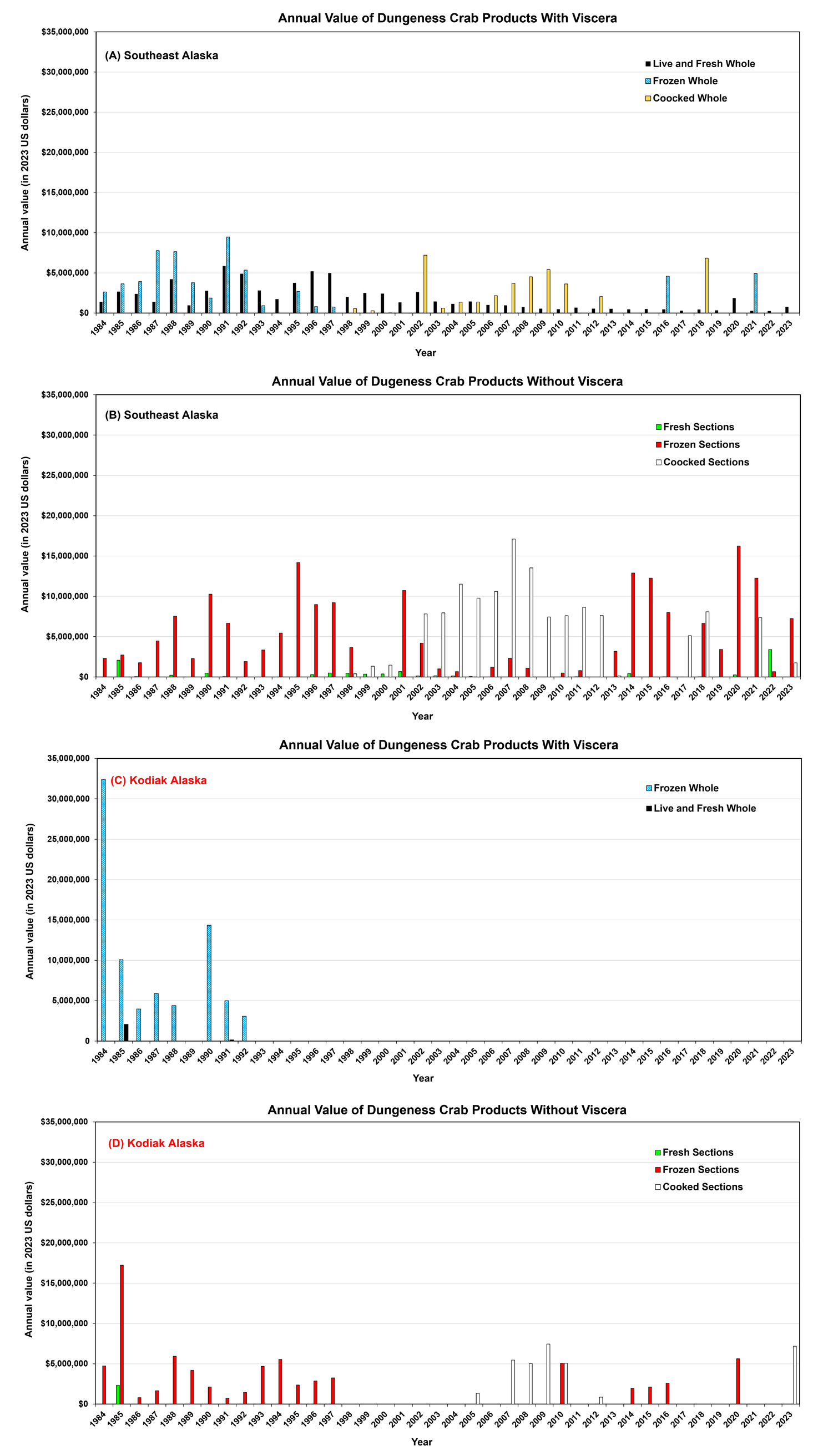

Supplement: S3 Fig — (A) The annual value of live and fresh whole, frozen whole and cooked whole crab products containing viscera processed in Southeast Alaska management area in 2023 inflation-adjusted dollars. (B) The annual value of live and fresh whole, frozen whole and cooked whole crab products without viscera processed in the Southeast Alaska management area in 2023 inflation-adjusted dollars. (C) The annual value of live and fresh whole, frozen whole and cooked whole crab products containing viscera processed in the Kodiak Alaska management area in 2023 inflation-adjusted dollars. (D) The annual value of live and fresh whole, frozen whole and cooked whole crab products without viscera processed in the Kodiak Alaska management area in 2023 inflation-adjusted dollars. Data source: Alaska Department of Fish and Game. (TIFF) [file pone.0330132.s003.tif]

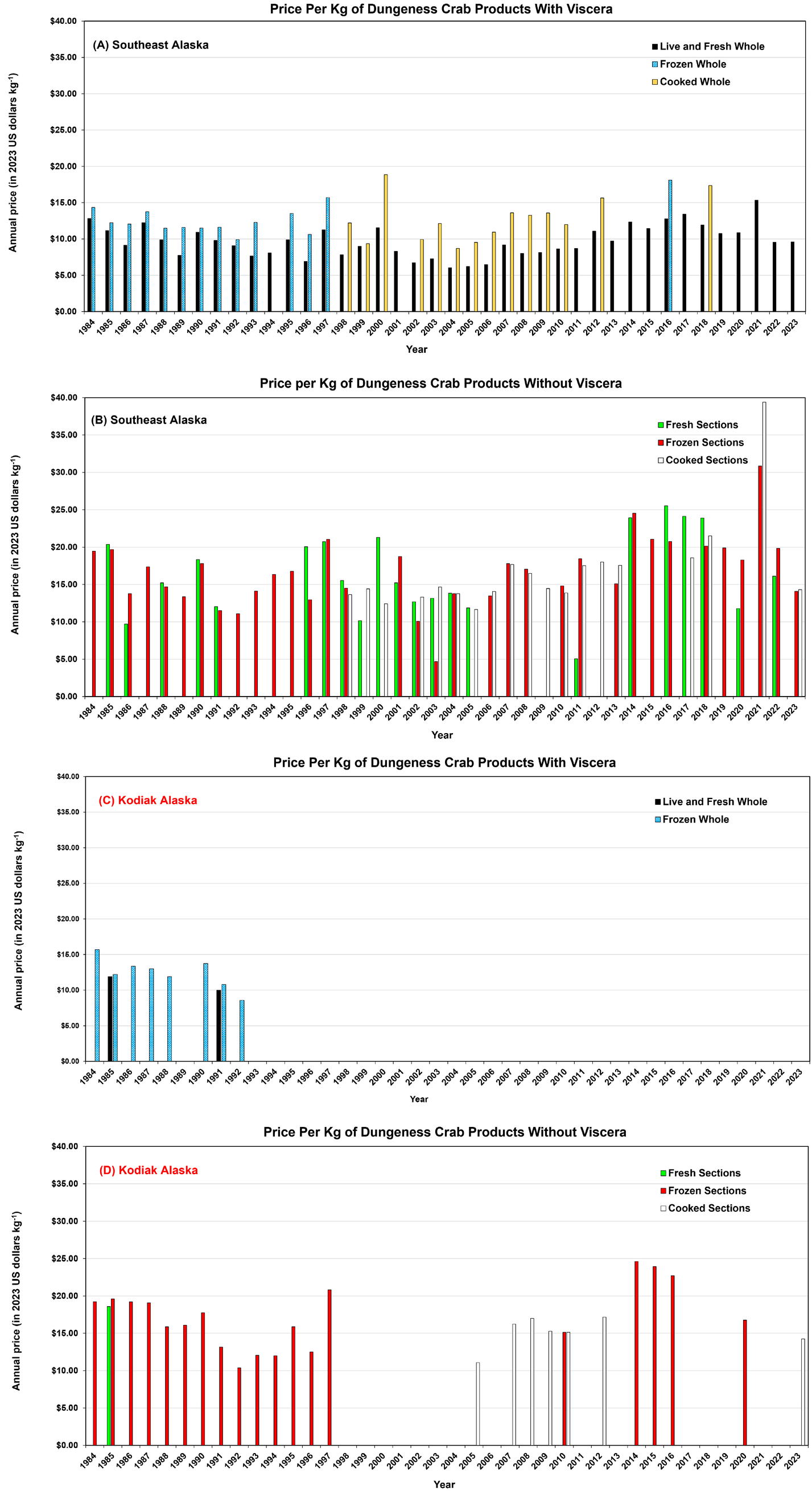

Supplement: S4 Fig — (A) The annual price kg-1 of live and fresh whole, frozen whole and cooked whole crab products containing viscera processed in the Southeast Alaska management area in 2023 inflation-adjusted dollars. (B) The annual price kg-1 of live and fresh whole, frozen whole and cooked whole crab products without viscera processed in the Southeast Alaska management area in 2023 inflation-adjusted dollars (C) The annual price kg-1 of live and fresh whole, frozen whole and cooked whole crab products containing viscera processed in the Kodiak management area in 2023 inflation-adjusted dollars. (D) The annual price kg-1 of live and fresh whole, frozen whole and cooked whole crab products without viscera processed in the Kodiak management area in 2023 inflation adjusted dollars. Data source: Alaska Department of Fish and Game. (TIFF) [file pone.0330132.s004.tif]

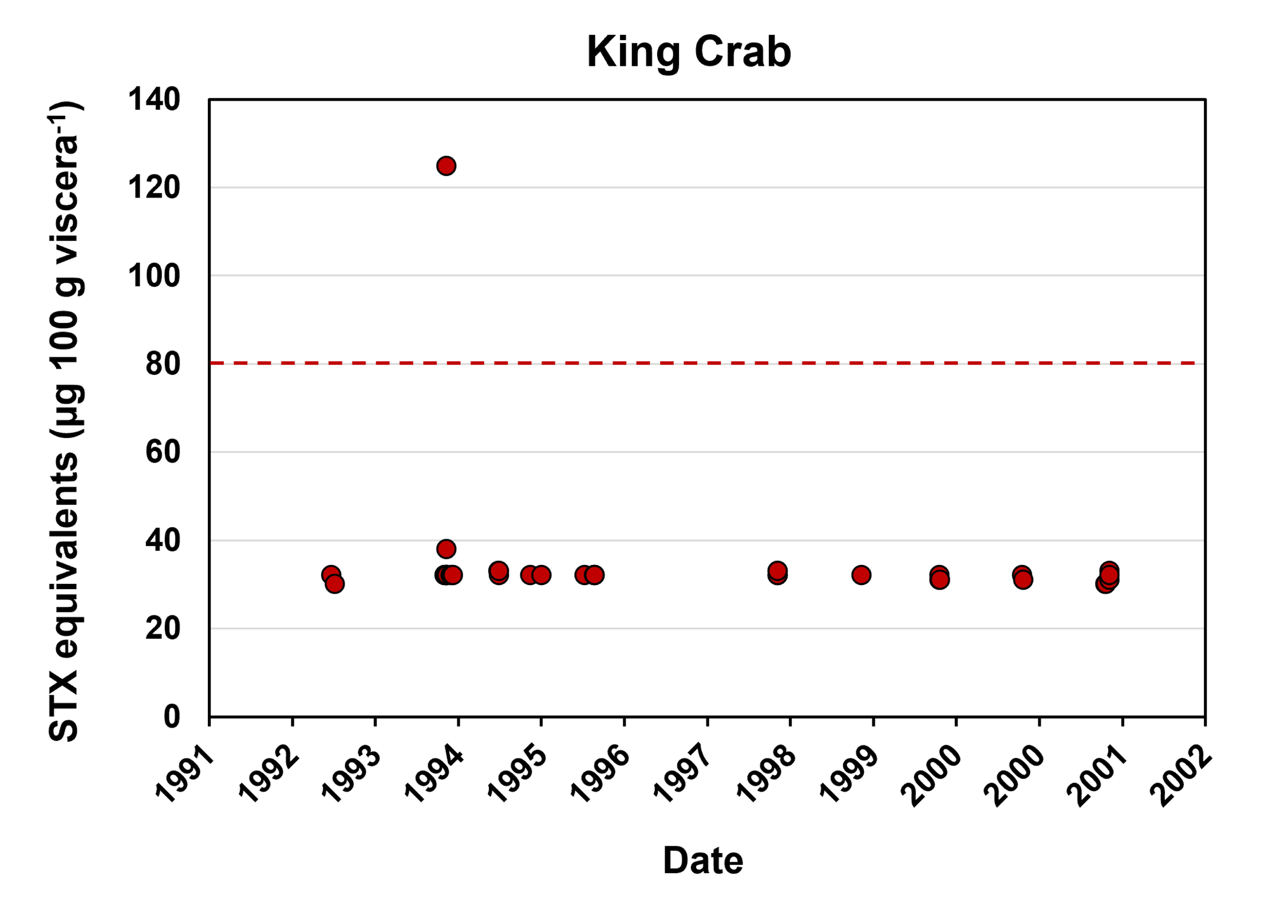

Supplement: S5 Fig — Samples were collected between 1992 and 2001. N = 40. The red line indicates the regulatory limit of 80 µg STX eq. 100 g viscera-1 for safe harvest. Data source: Alaska Department of Environmental Conservation – Environmental Health Laboratory. (TIFF) [file pone.0330132.s005.tif]
